# The Glasgow ‘Deep End’ Links Worker Study Protocol: a quasi-experimental evaluation of a social prescribing intervention for patients with complex needs in areas of high socioeconomic deprivation

**DOI:** 10.15256/joc.2017.7.102

**Published:** 2017-01-25

**Authors:** Stewart W. Mercer, Bridie Fitzpatrick, Lesley Grant, Nai Rui Chng, Catherine A. O’Donnell, Mhairi Mackenzie, Alex McConnachie, Andisheh Bakhshi, Sally Wyke

**Affiliations:** ^1^Institute of Health and Wellbeing, College of Medical, Veterinary and Life Sciences, University of Glasgow, Glasgow, UK; ^2^Institute of Health and Wellbeing, College of Social Sciences, University of Glasgow, Glasgow, UK; ^3^Urban Studies, School of Social & Political Sciences, Institute of Health and Wellbeing, College of Social Sciences, University of Glasgow, Glasgow, UK; ^4^Robertson Centre for Biostatistics, Institute of Health and Wellbeing, University of Glasgow, Glasgow, UK

**Keywords:** general practice, primary care, multimorbidity, complex interventions, health inequalities

## Abstract

**Background:**

‘Social prescribing’ can be used to link patients with complex needs to local (non-medical) community resources. The ‘Deep End’ Links Worker Programme is being tested in general practices serving deprived populations in Glasgow, Scotland.

**Objectives:**

To assess the implementation and impact of the intervention at patient and practice levels.

**Methods:**

*Study design*: Quasi-experimental outcome evaluation with embedded theory-driven process evaluation in 15 practices randomized to receive the intervention or not. *Complex intervention*: Comprising a practice development fund, a practice-based community links practitioner (CLP), and management support. It aims to link patients to local community organizations and enhance practices’ social prescribing capacity. *Study population*: For intervention practices, staff and adult patients involved in referral to a CLP, and a sample of community organization staff. For comparison practices, all staff and a random sample of adult patients. *Sample size*: 286 intervention and 484 comparator patients. *Outcomes*: Primary patient outcome is health-related quality of life (EQ-5D-5L). Secondary patient outcomes include capacity, depression/anxiety, self-esteem, and healthcare utilization. Practice outcome measures include team climate, job satisfaction, morale, and burnout. Outcomes measured at baseline and 9 months. *Processes*: Barriers and facilitators to implementation of the programme and possible mechanisms through which outcomes are achieved. *Analysis plan*: For outcome, intention-to-treat analysis with differences between groups tested using mixed-effects regression models. For process, case-study approach with thematic analysis.

**Discussion:**

This evaluation will provide new evidence about the implementation and impact of social prescribing by general practices serving patients with complex needs living in areas of high deprivation.

## Introduction

Health inequality is a global problem, and although it is driven by social determinants of health [1, 2], health and social care services can play an important role, at least in mitigating the effects of inequality [3]. Scotland has the widest health inequalities in western Europe [4], but attempts to reduce these have had limited success and are confounded by a perverse ‘inverse care law’, in which the complex health needs of patients living in deprived areas outstrip the availability of high-quality primary care [5, 6]. Patients in deprived areas also have a higher prevalence of multimorbidity, which occurs much earlier in life compared with those in affluent areas [7]. In deprived settings, patients present with multiple complex social, mental, and physical problems with which healthcare staff struggle to cope [8]. Due to high demand, consultation length is shorter than in affluent areas, and patients are less enabled (i.e. feel less able to cope with, understand, and manage their illness as a result of the consultation compared with patients in affluent areas) [9].

A recent policy response to inequalities has been the proliferation of different social prescribing schemes across the UK National Health Service (NHS). Social prescribing aims to expand the options available beyond what is traditionally provided in healthcare consultations by directing patients to local voluntary or community services that can offer patients support in terms of financial advice, work opportunities, and leisure and social activities. However, reviews have found that the evidence-base for the effectiveness of such approaches remains limited [10, 11]. A ‘links worker’ approach was identified in reports [12] produced by general practitioners (GPs) working in very deprived areas of Scotland (the ‘Deep End’ GPs), involving a collaboration of general practices serving the 100 most deprived populations in Scotland [13]. A similar model was previously explored in the Scottish Government funded Links Project [14], and in the BRIDGE (Building Relationships in Deprived General Practice Environments) project [15].

The Deep End paper, *‘What can NHS Scotland do to prevent and reduce health inequalities?*’, proposed practice-attached community links workers [16]. This proposal draws on two overlapping but distinct frameworks: social prescribing and asset-based community development; both of which are linked to a wider theory of community-oriented primary care [17]. The Deep End report argues that enabling patient engagement in community activities will enhance their community connection, trust, and cohesion, while reducing fear, suspicion, and intimidation. Within this context, the broad purpose of the proposed links worker was to “*act as a catalyst to hope and self-determination, using the strong relationships with patients that exist in general practice as a natural community hub*.” The rationale for this service development was that if individuals feel supported, they would be more likely to respond to information on ways to improve their health. It can also be seen as a response to the inverse care law, by providing practices and practitioners serving very deprived areas with an extra team member and a ‘horizontal’ referral pathway for patients, including those with complex multimorbidity spanning mental, physical, and social problems, which could potentially reduce GP and primary care workload.

Against this background, the Scottish Government funded the Glasgow ‘Deep End’ Links Worker Programme, which aims to support people living in the most deprived areas of Scotland to ‘live well’ in their communities. Since September 2014, seven general practices in areas of high social deprivation in the NHS Greater Glasgow and Clyde have been provided with: (i) a practice development fund and support to attend shared learning events; (ii) a practice-attached community links practitioner (CLP), an individual with community development work experience; and (iii) management support from programme staff. With these resources, the practice staff and CLPs can work with patients on non-medical problems through referral to local community organizations and, at the same time, develop practice capacity in this role and develop links with local community organizations. The conceptual and theoretical bases for the programme, as well as the operational and practical details, have been developed by GPs at the Deep End in collaboration with the professional body for GPs and third sector organizations.

The Links Worker Programme offers insight into the types of challenges likely to be encountered and the approaches that might be adopted by similar future programmes. Whilst there have been a number of ‘links-type’ initiatives in Scotland and elsewhere in the UK, the current evidence base provides little information about their impacts on patient or practice outcomes or on addressing health inequalities [18]. Consequently, there is a need for a robust evaluation of these ‘links’ approaches in order to assess their social and health impacts and their potential contribution to addressing health inequalities.

The aim of this evaluation is to provide evidence to inform future local and national programmes and policy decisions about the continuation and extension of the Links Worker Programme to practices serving deprived communities. The outcome evaluation is designed to test the hypothesis that a Links Worker Programme intervention will lead to improvements in the integration and use of community assets by practices, will enhance practice staff team morale and well-being, and will improve patients’ quality of life and well-being. The parallel process evaluation is designed to achieve an understanding of the theories of change that underpin the implementation of the intervention and of the barriers and facilitators affecting desired change.

### Protocol version and trial registration

This paper describes protocol version 1.1, dated 8 Jan­uary 2015. The trial is registered with the International Standard Randomised Controlled Trials (ISRCT): ISRCTN80842457. Any changes to the protocol will be approved by the Ethics Committee and notified to the Sponsor and Funder. Any changes affecting the information in the public trial register will be notified via the ISRCT.

## Methods/design and analysis

This is a quasi-experimental general-practice-level cluster randomized controlled trial with a parallel mixed-methods process evaluation. The design is based on the UK’s Medical Research Council framework for the evaluation of complex interventions [19]. It will use mixed quantitative and qualitative methods to assess the impact of the Links Worker Programme on a range of short-, medium-, and longer-term outcomes at the patient, practice, and community levels, and to determine the robustness, feasibility, and acceptability of the programme’s theories of change.

### Participants and setting

The study is based in 15 Glasgow general practices serving patients living in some of the most deprived areas in Scotland, including the practice of the Programme Clinical Lead, who submitted an application and practice development plan in response to an invitation to participate in the trial of the Links Worker Programme. The Programme Clinical Lead’s practice was selected to receive the intervention. The remaining 14 practices were then randomized by the Health and Social Care Alliance Scotland, which is charged with implementing and delivering the programme. Thus, the evaluation is based on data derived from seven intervention practices (i.e. the practice of the Programme Clinical Lead and six practices that were randomized to receive the programme resources) and eight comparator practices (i.e. those not randomized to receive the programme resources). All practices agreed to participate in this independent evaluation.

### Ethics and patient safety

This study was approved by the University of Glasgow College of Medical Veterinary and Life Sciences Ethics Committee (200140077). As participation in the ­evaluation has no associated risk to participants, no specific measures are applicable to determine patient safety.

### Inclusion and exclusion criteria

#### Practice staff

All staff in the intervention practices involved in delivery of the intervention and all staff in the comparator practices who would have been involved in the intervention had their practice been randomized to receive it. No exclusion criteria will be applied to either staff group.

#### Patients

Adult patients (aged 18 years or over) who are registered with an intervention practice and are referred (or self-referred) to a CLP during the study recruitment period, and a random sample of adult patients registered with a comparator practice. Patients will be excluded from the study by their usual medical/healthcare provider if, in the provider’s opinion, participation is contraindicated for health or social reasons (such as terminal illness or a family/other social crisis). Whilst reasons for such exclusions will be collected by the research team, identifiable information about patients will not. However, the provider will pass on the patients’ age, gender, and postcode (the latter to calculate deprivation from the Scottish Index of Multiple Deprivation).

#### Community staff

A convenience sample of staff from local community organizations will be recruited as a panel of key stakeholders. The organizations will be chosen with the help of the CLPs and practice staff on the basis of having had some engagement with practices and/or their patients.

### The intervention

The intervention comprises:

- A practice development fund (GBP 35,000 to spend on activities contributing to the development of seven ‘primary care team capacities’ (improvement in team well-being; shared learning within and between practices; increased awareness of patients’ needs; improved intelligence or understanding of local opportunities; signposting of patients to local resources; problem solving by the practice team; and network building by the practice team)
- A practice-attached CLP (an individual with community development experience employed by the Health and Social Care Alliance Scotland, but attached to the Practice)
- Management support from the programme (including support from the CLP’s line manager, the Programme Clinical Lead, Programme Manager, and the Programme Learning and Evaluation Officer. It also supports practices to send one GP and one Practice Manager to meet together six times over the duration of the trial to share learning).

### Recruitment of study participants

Study participant recruitment took place between March and December 2015, with the aim of completing all follow-up data collection by March 2017.

#### Intervention patient cohort

At the time of referral to a CLP, the healthcare provider gave patients the study information flyer and sought permission to pass on their contact details to the research team to discuss potential participation in the evaluation. In cases of self-referral, permission was sought by the CLP at the time of referral. When this permission had been obtained, a member of the research team telephoned patients to provide information about the evaluation and to obtain permission to mail them the study invitation pack (Participant Information Leaflet, Consent Form, questionnaire, and a pre-paid, pre-addressed envelope). On receipt of a completed questionnaire, patients were mailed a letter to acknowledge this with a GBP 5 gift voucher as a token of appreciation for their help. If there was no response 10 days after the mailing of the study materials, patients were telephoned again to determine their decision about participation, and if they expressed interest in participating they were given additional options for completing the questionnaire in either a face-to-face meeting or over the telephone with the study researcher. The aim was to collect baseline questionnaire outcome data prior to the start of the CLP intervention if at all possible. Patients who were not interested in participation were thanked for their time in considering the invitation and were reassured that the research team would not contact them again. For the process evaluation, a purposive sample of patients who consented to participate in the evaluation was invited to be interviewed.

#### Comparator practice patient cohort

Each comparator practice generated a list of a random sample of 1,000 patients on their register. The list was be reviewed by a GP principal in order to remove patients for whom contact was considered inappropriate. The practice then mailed the study invitation pack to the patients on the resultant list. On receipt of a completed questionnaire, patients were mailed a letter to acknowledge this with a GBP 5 gift voucher as a token of appreciation for their help. Patients who did not return a completed questionnaire were not followed up with any reminder telephone calls or mailings.

Patients in both the intervention and comparator practices were not denied any care that was available in the NHS during the study period.

#### Intervention and comparator practice staff

Study invitations packs were left with the practice manager to distribute to all eligible staff.

#### Community organization staff

For the intervention practices, staff from local community organizations were sent the study invitation pack. The aim was to recruit representation from two organizations on a panel of key stakeholders; other members included the practice manager and/or a lead GP, CLP, one other member of practice staff, and up to two patients involved in the practice developments (if possible). For comparator practices, the panel members consisted of the practice manager and/or a lead GP.

### Participant withdrawal

Patients who have completed the study baseline questionnaire but who subsequently decline or withdraw from the CLP intervention will revert to the usual care provided for other patients in their practice. Unless participants request to withdraw from the study, they will continue to be followed-up and will be analysed in the group to which the practice was allocated. If, however, any participant wishes to withdraw from the study, no further follow-up data will be requested, but data already provided will be used.

### Outcomes evaluation

#### Primary patient outcome

The primary patient outcome is health-related quality of life, measured at baseline and at 9-months’ follow-up by the EQ-5D-5L questionnaire [20] (Table 1). This widely used European tool has previously been used by the evaluation team in similar Deep End practices in Glasgow and found to be sensitive to change over a similar period of time to that of the present study [29].

#### Secondary patient outcomes

Secondary patient outcome measures at baseline and at the 9-month follow-up include the ICECAP-A measure [21], the Hospital Anxiety and Depression Scale [22], the Work and Social Adjustment Scale [23], Burden of Multimorbidity Measure [24], and self-reported lifestyle activities (smoking, alcohol, exercise) and healthcare utilization, including medication, hospital admissions, and GP consultations (Table 1).

#### Staff outcome measures

Staff outcomes at baseline and the 9-month follow-up include the Team Climate Inventory [25]; job satisfaction [26]; Morale Assessment in General Practice Index (MAGPI) [27]; and Maslach Burn-out Inventory [28] (Table 1). Knowledge of local community resources is assessed using questions devised and used in previous similar projects in Scotland. Demographic and socioeconomic variables were also collected at baseline.

#### Data collection

At baseline, data were collected on all primary and secondary outcomes as well as the number of long-term conditions and socio-demographic measures (age, gender, education, ethnicity, deprivation status [index of multiple deprivation based on postcode], and work status). These data will be collected again 9 months after recruitment, which is the primary outcome time point.

The primary method of self-reported data collection is via postal questionnaires; however, alternative completion methods, including telephone or home visits, were offered in order to maximize response rates. No identifiable data about patients will leave the practice unless patients have provided consent. All data are stored securely and confidentially at the University of Glasgow in line with its data-management policies.

#### Blinding

It was not possible to mask participants or healthcare professionals to the group allocation of their practice. It was also not feasible to blind members of the core study team collecting the data (B.F., L.G.), but the statisticians analysing the data (A.M., A.B.) and all other co-authors were blinded to the allocation. The statistical analysis plan was also reviewed and approved by the lead investigators, and by the senior study statistician, prior to the release of unblinded study results.

#### Sample size and analysis

No interim analysis is planned. Patient outcomes will be summarized as a whole and by intervention and comparison group at baseline and follow-up, and as changes over time. Analysis will be on an intention-to-treat basis using all available data. Differences between groups will be tested using appropriate mixed-effects linear or generalized linear regression models, allowing for clustering by practice and including an adjustment for the baseline value of the outcome measure. Since the comparator patients are respondents from a randomly selected sample, there are likely to be differences at baseline compared with the intervention patients, who are respondents from a group of patients referred to a CLP. Alternative methods will therefore be considered to control for selection bias, including adjustment for baseline factors (such as age, gender, deprivation, comorbidities). Tests for interactions will be used to identify subgroups that may benefit most from referral to the CLP.

The minimum target sample sizes of 286 patients for intervention and 484 patients for comparator practices is designed to have 80% power to detect a minimally important effect size of 0.274 standard deviation (SD) units in the EQ-5D-5L with a 95% degree of confidence, assuming an intra-class correlation of 0.01 and a 50% follow-up rate. This sample size will provide 90% power to detect an effect size of 0.316 SD units under the same assumptions. Analysis of staff outcomes will be descriptive as well as comparing total scores for each measure at baseline and follow-up in the intervention and comparison groups. We will also examine variation between practices within both groups. Study groups will be compared at baseline and at follow-up, and as changes over time using appropriate statistical methods.

### Process evaluation

The process evaluation comprises two phases.

#### Phase 1

Phase 1 developed and refined an effective theory of change (ToC) for the Links Worker Programme. The intention was to surface the ToC at the practice level; its anticipated processes and mechanisms through which change will be achieved; evidence for wider operationalization of the Self-Determination Theory (SDT) [30]; the programme’s predicted reach, engagement, and impact on health inequalities; short-, medium-, and long-term outcomes; contextual factors (local facilitators and barriers as well as those within the wider policy landscape) liable to impact on programme implementation and success in meeting its goals.

#### Phase 2

Phase 2 is guided by the RE-AIM framework [31] and aims to characterize Reach, Effectiveness, Adoption, Implementation and Maintenance of the Links Worker Programme (Table 2). In relation to ‘Reach’ and ‘Adoption’ at the patient level, the candidacy theory [32] will be used to: (i) explain the process through which people see themselves, and are seen by professionals, as ‘candidates’ for particular services; (ii) the way in which individuals and groups identify and engage with key stakeholders; (iii) how professionals decide whether a patient should be referred; (iv) the way in which those patients then negotiate the system and the impact of their previous healthcare experience; and (v) how policies and practices impact on what patients and professionals can do. In relation to ‘Effectiveness’, and explaining the process through which effects are produced, evidence will be sought on the extent to which the constructs of SDT (competencies, autonomy, and relatedness) underpin any changes observed in patients referred to the programme. In relation to ‘Implementation’ and ‘Adoption’ at the practice level, the Normalization Process Theory (NPT) [33] will be used to identify what helps and hinders the adoption and implementation of the programme in each practice. The NPT suggests that the likelihood that any complex new approach will ‘take hold’ in practices depends on: (i) how individuals understand and make sense of the intervention and its impact on others (coherence); (ii) the work that individuals have to do to engage others in enacting the intervention (cognitive participation); (iii) the work that different individuals or groups have to undertake to implement the new way of working, as well as the wider economic and policy resources which may support or hinder implementation (collective action); and (iv) how groups and individuals assess whether or not the intervention is working and how it might be modified (reflexive monitoring). Thus, it will provide a useful framework for understanding the progress made in each practice towards the embedding and routinization of the links worker approach. Different theories (candidacy, SDT, and NPT) have been chosen because no single theory is likely to answer fully the key questions within the RE-AIM framework. As outlined above, each theory offers a particular ‘lens’ through which to understand the different steps of RE-AIM.

#### Data collection

A ToC is essentially a comprehensive description and illustration of how and why a desired change is expected to happen in a particular context. It is focused, in particular, on mapping out or ‘filling in’ what has been described as the ‘missing middle’ between what a programme or change initiative does (its activities or interventions) and how these lead to desired goals being achieved. The original theories of change detailed in the programme documentation will be described and elaborated. The intervention and comparator practice plans will be reviewed in order to make explicit and compare their theories of change. These will be reviewed to comment on their likely plausibility, ‘doability’, and testability. However, to understand theories of change requires an understanding of the context in which organizations are operating. Two main types of contextual data will be considered: data on practice characteristics in relation to size and demographic profile; and data relating to the availability of potential community supports. Data relating to practice size and demographics will be accessed from the NHS Scotland Information Services Division (ISD) website using the most recent data available in February 2015:

- Age and gender data for the latest available quarter (1 July 2014)
- Deprivation data as at 30 September 2013
- GP data for the latest available quarter (1 July 2014).

Investigation of potential differences in availability of local community support services in areas served by intervention and comparison areas we will be based on two data sources: Infobase Team at the Glasgow Council for the Voluntary Sector (GCVS) [34], and A Local Information System for Scotland (ALISS) [35].

The data will be summarized in brief descriptions of what is delivered in each practice and any perceptions of impact. This will be mapped for both intervention and control practices – what is delivered to whom, by whom, and in what ways, and compare what is delivered to whom, by whom, and in what ways, and key differences between intervention practices and between intervention and comparison practices. Over time, further analysis will focus on addressing processes for establishing and maintaining the links and connections, and whether and how community-oriented approaches, including the Links Worker Programme, are sustained.

To further elaborate on sustainability of the programme and report on experiences and reactions of participants, individual interviews will be conducted with stakeholders in intervention practices towards the end of the implementation process. The interviews will gather perception of the reach and adoption of the programme at the patient level as well as experiences of its effectiveness and the processes through which these effects were achieved. The intention is to interview up to two patients, two managers of local community organizations, the CLP, a GP, and a member of reception staff or the practice manager. Interviews will focus on perceptions of sustainability of the programme, what has worked well and less well, other strengths and weaknesses of the approaches used, the perceived outcomes, and the characteristics of those most/least likely to benefit. The interview guide will again be guided, as appropriate, by the candidacy theory [32] and NPT [33], and will seek evidence for reference to the SDT [30]. As explained above, each theory offers a different lens through which to examine the success of the intervention based on the RE-AIM framework. The final phase of the analysis will be to integrate the findings from all the sources of data.

#### Analysis

Analysis of these data will be thematic, drawing on the Framework Approach [36]. With permission, interviews will be audio-recorded and transcribed verbatim. Thereafter, a coding framework will be developed based on repeated reading of the transcripts by the researcher supported by the evaluation team. The coding framework will be guided by questions posed in the research brief, by the NPT [33], candidacy theory [32], and SDT [30]. Descriptive data on experiences will be reported. Table 2 summarizes the elements of RE-AIM [31] and which theories are expected to help understand the processes in action at the different levels of the Links Worker Programme.

The use of three theories and one overall framework (RE-AIM) will help to generalize these findings in relation to other research on enabling system changes in general practice so as to reach people in further need of support, support people to take part in health-enhancing, community-based, activities, and implement the system changes.

### Study management and oversight

**Figure 1:**
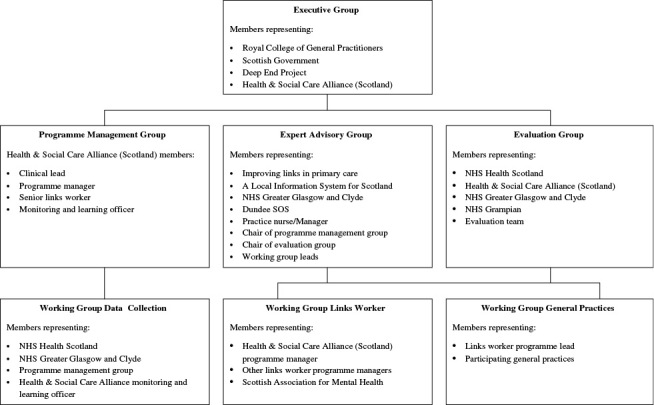
Programme governance and management. NHS, National Health Service; SOS, Sources of Support (SOS) social prescribing and community referral scheme.

The day-to-day management of the study is overseen by the evaluation management group, which feeds directly into the governance and management structure for the Links Worker Programme (Figure 1).

### Dissemination

Dissemination will include peer-reviewed publications and reports to healthcare professionals, commissioners, and policymakers. A summary report will also be sent to study participants, and linked to relevant websites such as the Scottish School of Primary Care, which is undertaking evaluations of new models of delivering primary care services.

## Discussion

The study will be a quasi-experimental outcome evaluation of a complex intervention comprising a links-worker approach to social prescribing, with embedded theory-driven process evaluation in 15 general practices randomized to receive the intervention (seven) or not (eight). This mixed-methods evaluation will provide useful evidence about the implementation and impact of a complex intervention designed to improve social prescribing and asset-based community development by general medical practices serving patients living in areas of high socio-economic deprivation in Scotland.

The findings of this study will help build on the very limited evidence-base for social prescribing [10, 11] in the context of the complex mix of physical, mental, social, and economic problems experienced by patients living in socially deprived areas [37], and the difficulties encountered by health professionals who care for them [8]. It will add to the evidence-base on multimorbidity generally [38], and in the context of deprivation specifically [7, 29]. Its findings will be timely in the present changing healthcare landscape as new models of care are being funded and piloted across the UK and elsewhere. If the findings suggest that this intervention does not have any ‘signal’ of a positive impact on patient or service outcomes, the implication would be that it might not be a worthwhile investment in general practices serving very deprived populations. If, on the other hand, the findings suggest a positive impact on either patient or service outcomes, further research would be required to assess whether or not these benefits are cost-effective. Unfortunately, a cost-effective analysis could not be included in the present study as this was not included in the funding award.

Whilst the research team has successfully conducted research using similar methods with similar patient populations, the present study poses some challenges relating to the likely achieved patient samples. In relation to the intervention practices, little is known about the patient caseload of CLPs in terms of the number, characteristics, or problems of new referrals received over any given time. Consequently, it is not possible to use any other strategy other than random sampling for the comparator practice patient sample. To allow statistical modelling to mitigate potential differences between the achieved samples, sampling of comparator practices will be based on approximately 20% of the adult patient population, i.e. 1,000 patients from each of the eight comparator practices.

The choice of quality of life as a primary outcome is a pragmatic one, as the intervention is generic and not single-disease focused and is in line with our previous approach [29]. This is deemed appropriate as quality of life is impaired greatly for patients with complex problems in deprived areas [39]. However, we will also be interested in the findings on all of the secondary outcomes, including mental health and health service utilization, such as number of GP consultations, given the exploratory nature of the study.

In terms of the design of the study and intervention, it should be emphasized that the research team was commissioned to evaluate the intervention almost a year after it started, and was not involved in its design, the randomization of practices, nor the implementation of the intervention. Weaknesses include the randomization methods, and the fact that some of the intervention and control practices are located in the same health centre, raising the possibility of contamination between groups. This will be explored in both the qualitative and quantitative analysis.

## Figures and Tables

**Table 1:** Outcome measures

Measure [reference]	Baseline	9-month follow-up
Primary patient outcome measure		
Quality of life (EQ-5D-5L) [20]	x	x
Secondary patient outcome measures		
ICECAP-A [21]	x	x
Hospital Anxiety and Depression Scale [22]	x	x
Work and Social Adjustment Scale [23]	x	x
Burden of Multimorbidity Measure [24]	x	x
Self-reported lifestyle	x	x
Service use	x	x
Staff measures		
Team Climate Inventory [25]	x	x
Job satisfaction [26]	x	x
Morale Assessment in General Practice Index (MAGPI) [27]	x	x
Maslach Burnout Inventory [28]	x	x

**Table 2:** RE-AIM: theories and level at which they may operate [31].

RE-AIM	Reach	Effectiveness	Adoption	Implementation
Relevant theory	Candidacy theory	Self-Determination Theory	Normalization Process Theory	Normalization Process Theory
Levels at which the Links Worker programme is expected to operate	Patient/professional	Patient	Patient Practice Community organization	Patient Practice Community organization
